# Susceptibilities of the Invasive Fall Armyworm (*Spodoptera* *frugiperda*) to the Insecticidal Proteins of Bt maize in China

**DOI:** 10.3390/toxins14080507

**Published:** 2022-07-24

**Authors:** Wenhui Wang, Dandan Zhang, Shengyuan Zhao, Kongming Wu

**Affiliations:** 1 State Key Laboratory for Biology of Plant Diseases and Insect Pests, Institute of Plant Protection, Chinese Academy of Agricultural Sciences, Beijing 100193, China; w975480209@163.com (W.W.); zhangdandan@bjfu.edu.cn (D.Z.); zhaosy90@126.com (S.Z.); 2 Guangdong Laboratory for Lingnan Modern Agriculture, Guangzhou 510640, China; 3 School of Grassland Science, Beijing Forestry University, Beijing 100083, China

**Keywords:** invasive fall armyworm, transgenic insect-resistant maize, Cry1Ab, Vip3Aa, susceptibility baseline, resistance management

## Abstract

To control the fall armyworm (FAW), *Spodoptera frugiperda* (Smith), a serious threat to maize production in China, the Chinese government has issued biosafety certificates for transgenic insect-resistant maize expressing Bt (*Bacillus thuringiensis*) toxins including Bt-Cry1Ab maize (crop event DBN9936), Bt-Vip3Aa maize (event DBN9501), Bt-(Cry1Ab+Vip3Aa) maize with superimposed traits (event DBN9936 × DBN9501) and Bt-(Cry1Ab+Vip3Aa) maize with superimposed traits (event Bt11 × MIR162), but the susceptibility baselines of geographically distinct FAW populations to these events, which form the basis for managing resistance development in the pest to these events, are not clear. We used the diet-incorporated bioassays method to detect the susceptibilities of the seven FAW populations collected from Yunnan, Henan and Hubei provinces in China in 2021 to the insecticidal proteins of the four Bt maize events. The result showed that the susceptibilities of different geographical populations to Bt insecticidal proteins were significantly different. In the seven populations, the range in median lethal concentrations (LC_50_) of Cry1Ab expressed in DBN9936 was 0.87–2.63 μg/g, 0.14–0.30 μg/g for Vip3Aa expressed in DBN9501, 0.78–1.86 μg/g for Cry1Ab+Vip3Aa expressed in DBN9936 × DBN9501, and 0.36–1.42 μg/g for CryAb+Vip3Aa expressed in Bt11 × MIR162. The growth inhibition responses also showed that the susceptibilities varied with the different median growth inhibitory concentration (GIC_50_) ranges (0.38–1.22, 0.08–0.28, 0.28–0.87, and 0.24–0.78 μg/g, respectively). The variations in the ranges of the susceptibility baselines of the geographical populations of fall armyworm in China to the insecticidal proteins expressed in the four events provide a scientific basis for monitoring FAW population resistance to Bt maize and managing the populations using different Bt maize events.

## 1. Introduction

Fall armyworm (FAW), *Spodoptera frugiperda* (Smith), is an omnivorous pest native to tropical and subtropical areas of the Americas and widely distributed on the American continent [1]. Since 2016, it has quickly invaded many countries in Africa and Asia and become a major pest of maize in these areas. FAW was first noted in China in December 2018 [2] and had spread to more than 20 provinces by 2019, damaging more than 1 million hectares. The Chinese government quickly established a nationwide monitoring and early warning system and an emergency prevention and control system to keep maize losses within 5% [3].

Chemical control is among the major tools for emergency prevention and control of FAW due to its efficient, rapid effect [4]. Organophosphorus and carbamate insecticides were mainly used to control FAW before 1980 [5]; since then, pyrethroid insecticides have been widely used [6]. In recent years, new insecticides such as emamectin benzoate, ethyl spinosad, and chlorantraniliprole have become the main controls [7]. However, the continuous use of any insecticide will inevitably lead to resistance. By 2017, FAW had already evolved resistance to at least 29 insecticidal active ingredients among six modes of action [8]. Resistance monitoring of FAW in China shows that the level of resistance to the newer insecticides is still relatively low, while the level of resistance to the traditional insecticides is medium to high [9]. Therefore, chlorantraniliprole, ethyl spinosad, and emamectin benzoate are mainly used to control FAW at present. Developing new control technologies to reduce the dependence on chemical insecticides is thus a critical need for the prevention and control of FAW.

Based on the global control strategy against FAW, planting transgenic insect-resistant maize is among the most economic and effective means. The United States began to plant Bt maize to prevent FAW in 1996, with 33.17 million hectares planted with Bt maize by 2019 [10]. FAW occurrence and damage and the quantity of chemical insecticides have been significantly reduced. However, FAW has evolved resistance to a variety of Bt maize lines. The resistance ratios of FAW reported in 2010 in Puerto Rico to the Cry1F insecticidal protein expressed in the TC1507 event were likely in excess of 1000: 1 [11], so TC1507 was withdrawn from the local market [12]. Substantial resistance to Cry1F was also reported for Brazil, Florida, and North Carolina during 2014–2016 [13,14,15]. Based on monitoring in 2010 to 2015, FAW in Brazil and Argentina had developed resistance to event MON810 expressing Cry1Ab insecticidal protein, and its field efficacy against FAW was reduced significantly [16]. At present, the resistance allele in FAW to Vip3Aa20 protein has been detected in Brazil, but at low frequency in the population [17]. Clearly, resistance monitoring and management is vital for successful control of FAW in regions planted with Bt maize.

For using Bt maize to control FAW and reduce dependence on chemical insecticides in China, the Chinese government issued safety certificates for production and application of Bt maize events used in this study, such as DBN9936, DBN9501, DBN9936 × DBN9501, and Bt11 × MIR162. These events have provided high efficacy against invasive FAW in field experiments, but the susceptibilities of geographically distinct FAW populations to these events are not clear. Toward constructing a resistance monitoring and management system for FAW in China, we here determined the susceptibilities of seven geographic populations to the toxins expressed in four events and established the resistance monitoring baselines of the invasive FAW.

## 2. Results

### 2.1. Dose Responses of Larvae from Different FAW Populations to Bt Toxins in Maize

Among the FAW populations, the LC_50_ for DBN Cry1Ab ranged from 0.87 to 2.63 μg/g, the LC_50_ for DBN Vip3Aa ranged from 0.14 to 0.30 μg/g, the LC_50_ for DBN Cry1Ab+Vip3Aa ranged from 0.78 to 1.86 μg/g, and the LC_50_ for Syngenta Cry1Ab+Vip3Aa ranged from 0.36 to 1.42 μg/g (Table 1). The fold difference between the most-susceptible and most-tolerant populations for each of the respective toxins above was 3.02, 2.14, 2.38, and 3.94. The LC_95_ for DBN Cry1Ab ranged from 19.13 to 39.12 μg/g, the LC_95_ for DBN Vip3Aa ranged from 0.44 to 3.73 μg/g, the LC_95_ for DBN Cry1Ab+Vip3Aa ranged from 12.53 to 26.95 μg/g, and the LC_95_ for Syngenta Cry1Ab+Vip3Aa ranged from 2.13 to 10.26 μg/g. The LC_50_ for the same insecticidal protein differed significantly among the populations. The LC_50_ value for DBN Cry1Ab in the Xinyang population was significantly higher than in the others and did not differ between the Xinxiang and Ezhou populations, but the LC_50_ for these two populations was significantly higher than for the Xundian and Puer populations. The LC_50_ for DBN Vip3Aa was significantly higher for the Dehong and Zhoukou populations than those in Xinyang, Xundian and Ezhou populations, and the difference among the other populations was not significant. The LC_50_ value to DBN Cry1Ab+Vip3Aa of Xinyang was significantly higher than that of other populations except Dehong population. The difference between the LC_50_ for Dehong and Xinxiang was not significant, but was significantly higher compared with those for the Puer and Xundian populations. The LC_50_ for Syngenta Cry1Ab+Vip3Aa of the Xinyang population was significantly higher than that of the Zhoukou, Puer, Ezhou, and Xundian populations.

**Figure 1:**
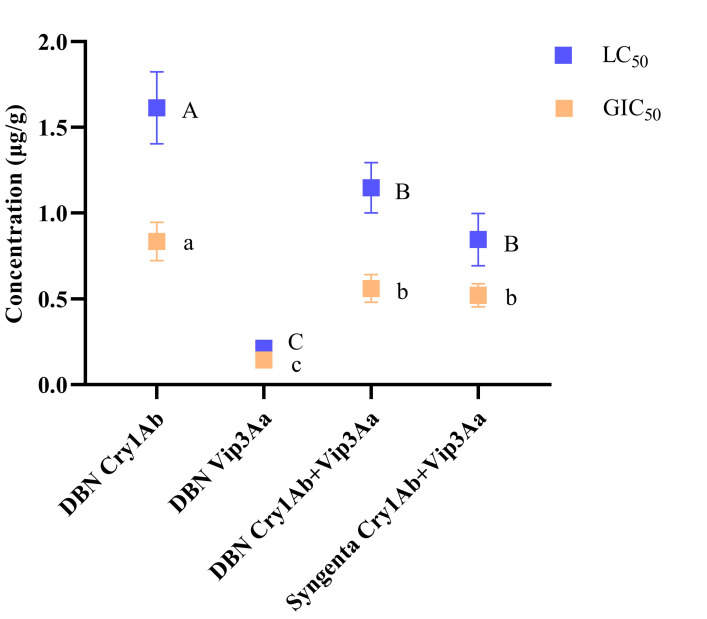
Mean (±SE) of LC_50_ and median growth inhibitory concentration (GIC_50_) of seven populations for different Bt insecticidal proteins. Different uppercase letters indicate significant differences among the LC_50_ for different Bt insecticidal proteins (*p* < 0.05, Duncan’s test); different lowercase letters indicate significant differences among the GIC_50_ for different Bt insecticidal proteins (*p* < 0.05, Duncan’s test).

The average LC_50_ values of seven populations to a toxin were compared (Figure 1), the LC_50_ values for DBN Cry1Ab (1.61 ± 0.30 μg/g) in all populations was significantly higher than for the other three insecticidal proteins (*p* < 0.05). The values for DBN Cry1Ab+Vip3Aa (1.15 ± 0.21 μg/g) and Syngenta Cry1Ab+Vip3Aa (0.85 ± 0.22 μg/g) did not differ significantly (*p* > 0.05), but were significantly higher than for DBN Vip3Aa (0.21 ± 0.04 μg/g) (*p* < 0.05). LC_50_ values in the populations from high to low were DBN Cry1Ab > DBN Cry1Ab+Vip3Aa and Syngenta Cry1Ab+Vip3Aa > DBN Vip3Aa.

### 2.2. Dose Responses of Larvae Mass to Bt Proteins from Maize in Different FAW Populations

The range in GIC_50_ values among the FAW populations for DBN Cry1Ab was 0.38–1.22 μg/g, the range in GIC_50_ values for DBN Vip3Aa was 0.08–0.28 μg/g, the range in GIC_50_ values for DBN Cry1Ab+Vip3Aa was 0.28–0.87 μg/g, and the range in GIC_50_ values for Syngenta Cry1Ab+Vip3Aa was 0.24–0.78 μg/g (Table 2). Populations differed in susceptibility for the respective proteins by 1.66 to 3.21 fold, 1.13 to 3.50 fold, 1.43 to 3.11 fold, and 1.63 to 3.25 fold. The GIC_95_ values ranged from 4.04 to 7.38 μg/g for DBN Cry1Ab, 0.25 to 0.88 μg/g for DBN Vip3Aa, 2.14 to 5.91 μg/g for DBN Cry1Ab+Vip3Aa, 1.11 to 2.57μg/g for Syngenta Cry1Ab+Vip3Aa. There were also significant differences in GIC_50_ among the populations tested for the same insecticidal protein. The GIC_50_ value for DBN Cry1Ab did not differ significantly among the Ezhou, Xinyang, and Dehong populations, but was significantly higher for these three than for the Xundian population. The GIC_50_ value for DBN Vip3Aa in the Dehong population was significantly higher than in other populations and in the Zhoukou compared to the Ezhou population, whereas the difference between those for the Puer and Xinxiang populations was not significant, but significantly higher than for Xundian population. For DBN Cry1Ab+Vip3Aa, GIC_50_ values did not differ significantly between the Ezhou and Xinyang populations, but were significantly higher for these two compared with the other populations except Xinxiang, and the difference was not significant among the other populations. The GIC_50_ value for Syngenta Cry1Ab+Vip3Aa of Xinyang population was significantly higher than that of other populations except Xinxiang, Puer and Zhoukou had no significant difference but was significantly higher than Xundian population, and the difference between Dehong and Ezhou was not significant.

The GIC_50_ values for the different Bt proteins differed significantly among the FAW populations (Figure 1). The GIC_50_ for DBN Cry1Ab (0.83 ± 0.16 μg/g) was significantly higher than that for the other three insecticidal proteins (*p* < 0.05), and the difference between the values for DBN Cry1Ab+Vip3Aa (0.56 ± 0.11 μg/g) and Syngenta Cry1Ab+Vip3Aa (0.52 ± 0.09 μg/g) was not significant (*p* > 0.05), but they were significantly higher than those for DBN Vip3Aa (0.14 ± 0.04 μg/g) (*p* < 0.05). GIC_50_ values from high to low were in the order of DBN Cry1Ab > DBN Cry1Ab+Vip3Aa and Syngenta Cry1Ab+Vip3Aa > DBN Vip3Aa.

**Figure 2:**
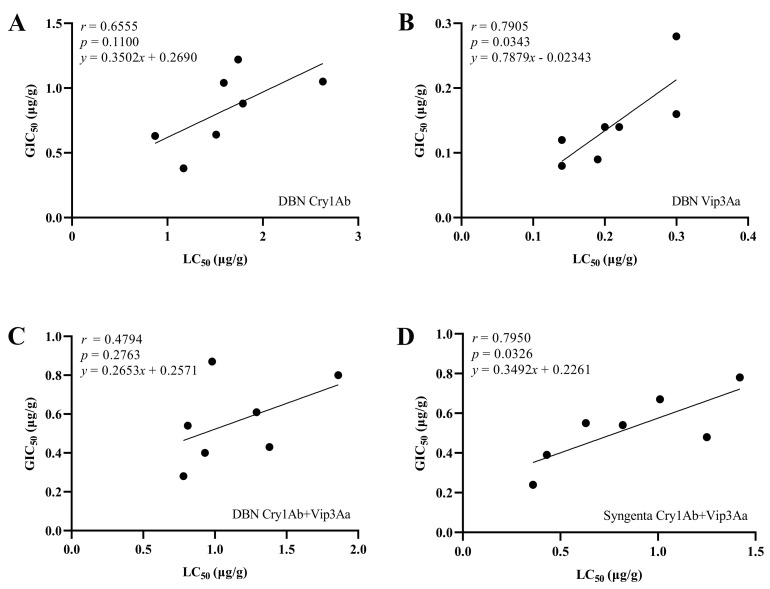
Correlation between the LC_50_ and GIC_50_ for insecticidal proteins from different Bt maize events. (**A**) DBN Cry1Ab; (**B**) DBN Vip3Aa; (**C**) DBN Cry1Ab+Vip3Aa; (**D**) Syngenta Cry1Ab+Vip3Aa.

The correlation analysis showed that there was no significant correlation between LC_50_ and GIC_50_ for DBN Cry1Ab, DBN Cry1Ab+Vip3Aa on FAW (*p* > 0.05); However, the LC_50_ and GIC_50_ values for DBN Vip3Aa and Syngenta Cry1Ab+Vip3Aa were moderately correlated among the FAW populations (*p* < 0.05, *r* = 0.7905 and 0.7950, respectively) (Figure 2), indicating that the lower the LC_50_, the stronger the inhibition of larval development, and the higher the susceptibility of FAW to the insecticidal protein.

## 3. Discussion

Previous studies have shown that the Bt maize in China had high efficacy against FAW. Bt-Cry1Ab and Bt-(Cry1Ab+Vip3Aa) maize were highly toxic to the larvae. The mortality of FAW larvae that feed on Bt-Cry1Ab maize leaves is less than 65% and 53.02 to 100% for those that feed on Bt-(Cry1Ab+Vip3Aa) maize leaves, and growth and development of any surviving larvae is significantly inhibited [18]. The mortality of FAW larvae that feed on different tissues of Chinese Bt-Cry1Ab maize DBN9936 ranges from 34 to 100% [19]. Bt maize is a potential means to control FAW in China with good commercial prospects.

Our results indicated low interpopulation variations in susceptibilities to the insecticidal proteins of the four Bt maize events. The ranking for lethality and growth inhibition of FAW by the Bt proteins expressed in the four Bt maize events to the invading FAW (DBN Vip3Aa > Syngenta Cry1Ab+Vip3Aa > DBN Cry1Ab+Vip3Aa > DBN Cry1Ab) indicates that the greater susceptibilities of FAW to Bt-(Cry1Ab+Vip3Aa) maize with superimposed traits and Bt-Vip3Aa maize than to Bt-Cry1Ab maize. Thus, planting bivalent Bt maize expressing Vip3Aa will be more effective against FAW.

Our results also showed that the Bt protein levels expressed in seedling stages of the four Bt maize events were higher than the LC_95_ values of FAW populations, which meant the Bt maize had a high efficiency for control of the pest. However, there is a basic principle that the levels of Bt proteins in maize gradually decrease as its growth, especially in the late stage of maize, the levels of Bt proteins dropped quickly [19]. Therefore, it is necessary to take a detail study to understand seasonal change of Bt toxin in each Bt variety planted.

In previous measures of the susceptibilities of FAW to different Bt proteins. Li et al. (2019) found that the order of lethal effect of five Bt proteins to the FAW population invading Yunnan was: Vip3Aa > Cry1Ab > Cry1F > Cry2Ab > Cry1Ac [20]. The lethal effects of Vip3Aa and Cry1Ab were consistent with the results of our study, indicating that planting Bt maize expressing Cry1Ab, Vip3Aa, or superimposed Cry1Ab and Vip3Aa can effectively control the occurrence and damage of FAW. In addition, it has been reported that the susceptibilities of FAW to Bt proteins varied among different populations in Brazil. The median effective concentration (EC_50_) of Cry1Ab in the FAW field populations in Brazil ranged from 0.30 to 3.67 μg/mL and had a 12-fold difference among several populations [16]. The LC_50_ of Vip3Aa20 changed from 92.38 to 611.65 ng/cm^2^ and presented a 6.6-fold difference among populations, indicating that natural variation was responsible, not variation due to selective pressure as a result of past exposures [21]. In the present study, the susceptibilities of the FAW populations in China to the insecticidal proteins expressed in the four maize events might be related to the types of chemical insecticides used for emergency prevention and control of FAW. The long-term use of *Bacillus thuringiensis* or compound insecticides containing *B. thuringiensis* toxins [22] in China may indirectly increase the resistance of FAW to Cry1Ab, thereby reducing the susceptibility of FAW to monovalent or bivalent Bt maize expressing Cry1Ab protein. At the same time, invasive FAW can also feed on cotton in addition to corn [23]. Due to the spatiotemporal overlaps between maize planting regions and the main cotton-producing areas in China, FAW that feed on Cry1Ac cotton may increase the risk of resistance developing in Bt-Cry1Ab maize.

To ensure long-term, effective control of FAW using Bt maize, resistance management must include (1) establishing a baseline for the susceptibilities of FAW to the corresponding Bt insecticidal proteins before commercialization of the Bt maize and (2) implementing a strict high dose/refuge strategy and (3) thoroughly monitoring resistance when Bt maize is used. For example, before issuing the registration certificates for Bt crops, the United States requires the registrant or company to submit the susceptibilities of target pests to Bt proteins, such as the lethal dose and mortality and provide a field resistance monitoring plan, shelter implementation plan and a grower education and training plan after registration [24].

At present, the most widely used resistance management strategy in the world is the high-dose/refuge strategy [25,26], which has been successful in the United States for more than 20 years [25]. Three key points are required: (1) Bt crops must express a high dose of proteins that are insecticidal to the target pests; (2) the initial resistance allele frequency in the target pests is low (<0.001); (3) set up sufficient non-Bt crop refuges [27]. A high dose is defined as the amount of protein expressed in insect-resistant maize plants that can kill 100% of sensitive homozygous individuals (SS) and 95% of sensitive heterozygous individuals (SR) in the target pest populations [25]. Refuge refers to all non-Bt plants planted near Bt crops that can enable the normal growth and development of target pests. There are three types of refuges: (1) structural refuge with a certain proportion of non-Bt maize is planted near Bt maize to reduce the speed of resistance development in FAW; (2) seed mixture refuge, with a certain proportion of non-Bt maize seeds mixed in the commercial Bt seeds; (3) natural refuge of weeds or cultivated plants that can provide sensitive pests near Bt maize [28]. Refuges can dilute the resistance gene of field populations, prolong the service life of Bt maize, and delay the development of resistance of FAW.

Due to different planting structures and patterns in different countries, the resistance management strategies are also slightly different. The United States and Canada mainly use structural refuges and seed mix refuges to delay the resistance of target pests [29]. The prevention and control of lepidopteran pests by single-gene Bt maize requires the planting of 20% structural refuge [11,12]. However, because the FAW is more widespread in the southern United States than in the northern, the refuge area is also greater. Argentina and Brazil in South America uniformly require the planting of 10% structural refuges [16,30,31].

Although the high-dose strategy combined with the refuge strategy has been implemented in the world for more than 20 years, different countries have achieved different results. The intensive production mode in developed countries including the United States has high requirements for refuges, while the refuge strategy is difficult to implement in the small-scale production mode in developing countries and target pests rapidly develop resistance [27]. The strict implementation of refuge requirements for Bt maize growers and high compliance rate in the United States (>85%) and Canada (>80%) has greatly contributed to the long-term success of Bt maize in North America. However, resistance of FAW developed rapidly in Brazil because it is difficult to implement refuge requirements among Brazilian growers, with <20% compliance [13,32].

Drawing on the experience of other countries controlling the resistance of target pests of Bt maize, China should adopt a strategy using “Natal Source insect resistance management (IRM)” and high-dose/refuge suitable for our conditions based on the biological characteristics, host damage, migration and dispersion of FAW in maize regions, and effectively implement the strategy. At the same time, long-term, accurate monitoring of resistance in field populations of FAW is needed to provide a scientific basis for evaluating the effectiveness of the resistance management measures [33]. Before Bt maize is commercialized, a high-dose evaluation system for Bt maize against FAW first needs to be established to ensure that the Bt maize lines express the insecticidal proteins in high doses that kill target pests in their source areas. At the same time, baselines for the susceptibility of FAW populations to the insecticidal proteins expressed in the candidate Bt maize lines should be determined as a reference for monitoring population resistance after commercialization. Secondly, a regional resistance management strategy needs to be formulated and implemented in combination with the maize planting regions and the FAW infestation areas in China [33]. The Southwest Hilly Corn Region and the Southern Hilly Corn Region in China are the immigration and winter breeding areas for overseas FAW [34], and the source of FAW in the Huanghuaihai Summer Corn Region and the Northern Spring Corn Region. Because generations of FAW overlap and maize is mostly planted twice each year, FAW will face high selection pressure if Bt maize is planted in this area, thus increasing the risk of resistance [33]. Therefore, when planting Bt maize in this area, we should expand the size of the refuge and reduce the selection pressure from Bt maize to prevent the northward migration of FAW that carry resistance genes into other maize production areas. At the same time, a multi-gene strategy should be followed to avoid planting events that express a single gene, especially Bt maize that only expresses the Cry1Ab protein [33]. Multivalent Bt maize with high-dose expression of Cry1 and Vip3A should be planted to control the number of FAW at their source. Bt maize that expresses a single Cry1 class should not be planted in the Huanghuaihai summer maize region, as shown by the refuge strategy to control cotton bollworm in the Yellow River Valley [35], using natural refuges such as soybeans and sorghum to delay the resistance development of FAW. Other maize production areas are mainly located in FAW immigration areas, so the appropriate Bt maize event should be selected according to the pest species present. In addition, because farming in China is a small-scale mode, farmers should bear the main responsibility for implementing the refuge requirements when planting Bt maize, while the government is responsible for guidance and supervision. Based on the current research and development status of Bt maize in China and the maize production mode, a 10–20% structural refuge should be required to control FAW [36]. After the commercialization of Bt maize, long-term monitoring of the dynamics of resistance development of FAW field populations in different maize regions is required using existing Bt crop resistance monitoring technologies such as LC_50_, diagnostic concentration, single pair crossing, F_2_ screening, and molecular methods [37] to develop an early warning system for FAW resistance. Finally, the monitoring and early warning system for FAW must be improved by accurately monitoring the FAW migration dynamics with insect radars, geographic information systems, and global positioning technology, and placing light traps and food traps in the migration areas and routes and landing areas to reduce adult densities to the greatest extent. All these approaches are needed to create an effective ecofriendly, sustainable prevention and control system with insect radar monitoring and Bt maize as the core of the system to improve resistance management [38].

Because resistance monitoring is the basis for managing Bt maize resistance in FAW, establishing baseline data on the susceptibility and frequency of resistance alleles in FAW field populations of FAW to Bt insecticidal proteins is crucial. The baseline data forms the foundation for the design and implementation of a reliable resistance monitoring program. The present study provides these susceptibility baselines for FAW populations in the main infestation and immigration areas in China to the insecticidal proteins expressed in the Bt maize events that received biosafety certificates. Our data also provide a scientific basis for formulating effective resistance management measures in China.

## 4. Conclusions

Herein, we tested different geographical populations of fall armyworm in China to elucidate the ranges in the susceptibility baselines to the insecticidal proteins expressed in four Bt maize events with biosafety certificates. The results showed low interpopulation variations in FAW susceptibilities to the insecticidal proteins of the four Bt maize events. The susceptibility of the FAW populations to the insecticidal proteins expressed in Bt-(Cry1Ab+Vip3Aa) maize and Bt-Vip3Aa maize was higher than for Bt-Cry1Ab maize. These susceptibility baselines can now be used as a reference to monitor and manage resistance in FAW populations after these four Bt events are commercialized in China and thus maintain their effectiveness.

## 5. Materials and Methods

Insecticidal proteins were lyophilized powder of seedling leaves of each Bt maize event, and FAW populations were collected from maize fields in the main areas affected by FAW in China.

### 5.1. Transgenic Insect-Resistant Maize Events and Insecticidal Proteins Expressed

Bt-Cry1Ab maize (“DBN9936” event), Bt-Vip3Aa maize (“DBN9501” event), Bt-(Cry1Ab+Vip3Aa) maize (“DBN9936” event × “DBN9501” event) and negative control maize line (Nonghua 106) were provided by the DBN Group, Beijing. Bt-(Cry1Ab+Vip3Aa) maize (“Bt11” event × “MIR162” event) and negative control maize line (Xianda 901) were provided by Syngenta, Beijing. All maize varieties were potted (5 plants/pot) in an air-conditioned greenhouse (about 26 °C). As plants reached the stage with 4 leaves and 1 shoot (plant height about 30 cm), all plant leaves of each variety were removed, placed in a sealed plastic freezer bag, and then moved to an ultrafreezer at −80 °C for 12 hours, then freeze-dried (approximately 48 hours). The samples were then ground to a fine powder using a tissue grinder. Ground samples for an event were mixed thoroughly and subdivided into 50 mL centrifuge tubes and kept at −80 °C until use.

A sandwich enzyme-linked Immunosorbent assay (ELISA) was used to quantify proteins; the Cry1Ab/Cry1Ac Quantiplate Kit (Envirologix, Portland, ME, USA) was used for Cry1Ab proteins and the Vip3A Quantiplate Kit (YouLong Biotech, Shanghai, China) for Vip3Aa proteins according to the manufacturer’s instructions. The Wash Buffer + Tween-20 was added to the sample at a ratio of 1 mg sample to 100 μL buffer, then the sample was mixed with shaking overnight. The samples were centrifuged at 12,000 rpm for 15 min, and the supernatant was collected and diluted into suitable concentrations for ELISA assays. This was repeated 3 times for each well and the optical density (OD) values were read at 450 nm with the Synergy H1 Microplate Reader (Biotek, Winooski, VT, USA). Data were subjected to a simple regression analysis in Excel 2010 (Microsoft, Redmond, WA, USA). The results are in Table 3.

### 5.2. Collection and Breeding of FAW

FAW populations were collected from Yunnan Province, the main area of occurrence, and Hubei and Henan provinces, where FAW arrived in 2021. Detailed information is given in Table 4. The larvae were fed an artificial diet based on soybean powders and wheat bran [39]. The artificial diet was cut into small pieces. Each well in a 24-well plate contained one piece of the diet and one larva. When the larvae grew to the 5th instar, they were transferred to a glass tube with artificial diet that was then closed with a cotton plug for pupation and adult emergence. The adults were placed in cages (4 × 25 × 25 cm), and fed a 10% *w*/*v* sugar solution to supplement nutrition and water. The top of each cage was covered with white medical gauze for oviposition, which was changed every 24 h and put into a zip lock bag with any eggs. The resulting neonates were placed in a climate-controlled insect chamber at 26 ± 1 °C, 60 ± 10% relative humidity (RH) with a photoperiod of 16 h: 8 h (L: D) and used for bioassays within 12 h after hatching.

### 5.3. Bioassay of FAW Susceptibilities to Bt Proteins

Bt protein samples from Bt maize leaves were diluted with artificial diet. The toxins were tested at the following concentrations: 0.3827, 0.7654, 1.5308, 3.0616, and 6.1232 μg/g DBN Cry1Ab; 0.1016, 0.2032, 0.4064, and 1.0160 μg/g DBN Vip3Aa; 0.4064, 0.8128, 1.6256, 3.2512, and 6.5024 μg/g DBN Cry1Ab+Vip3Aa; 0.5574, 1.1148, 2.2296, 4.4592, and 8.9184 μg/g Syngenta Cry1Ab+Vip3Aa (μg/g: μg of Bt protein per g of artificial diet). Corresponding amounts of the negative control samples were mixed with the artificial diet as control. The diluted toxin diets (0.5 g) were added with one neonate FAW to each well of 24-well plates in a completely randomized, with 3 replicates per concentration and 48 larvae per replicate at each concentration for a total of 144 larvae per concentration. The plates were then placed in an insect chamber. Depending on the freshness of the diet and amount consumed, the diet was either replaced or more was added during the 14 d assay. After 14 days, we counted any dead larvae; any larva that did not crawl normally when touched with a brush was deemed dead. We then calculated the mortality and corrected mortality, weighed each larva in the control group and the treatment group, and calculated the growth inhibitory rate.

### 5.4. Statistical Analyses

Corrected mortality and growth inhibitory rates of FAW larvae were calculated as:

Corrected mortality (%) = (treatment group mortality − control mortality)/(1 − control mortality) × 100(1)

Growth inhibitory rate (%) = [(body mass increase in control group − body mass increase in treatment group)/body mass increase in control group] × 100(2)

The susceptibilities of different populations to Bt toxins were analyzed by probit regression using SAS 9.4 (SAS Institute, Cary, NC, USA), which generated LC_50_ values with 95% fiducial limits (FL), chi-square (χ^2^), slope with standard errors (slope ± SE), and degrees of freedom (*df*). The difference between LC_50_ values and LC_95_ values in diet-incorporated bioassays was considered significant if the 95% fiducial limits of the values did not overlap. The estimates of GIC_50_ and GIC_95_, their respective confidence intervals, and the mass of all larvae at each concentration were evaluated using a nonlinear regression analysis.

A one-way ANOVA was used to compare significant differences among the LC_50_ or GIC_50_ for the different insecticidal proteins; Duncan’s multiple-range test was used for multiple comparisons to identify the means that differed. The LC_50_ and GIC_50_ for different geographical populations were analyzed for correlations using SPSS 23.0, and the correlation coefficient *r* and *p* were calculated. SPSS 23.0 (IBM, Armonk, NY, USA) was used for these analyses.

## Figures and Tables

**Table 1:** Mean lethal concentrations of insecticidal proteins expressed in Bt maize against FAW larvae from different populations in China.

Bt Event	Protein	Population	*N*	LC_50_ (95% FL) µg/g	LC_95_ (95% FL) µg/g	Slope ± SE	χ^2^	*df*	*p*
DBN9936	DBN Cry1Ab	Xundian	720	1.17 (0.95–1.42) c	32.09 (18.51–72.54) a	1.14 ± 0.12	0.71	3	0.87
		Dehong	720	1.59 (1.31–1.92) bc	39.12 (22.32–89.25) a	1.18 ± 0.12	6.21	3	0.10
		Puer	720	0.87 (0.67–1.08) c	29.75 (16.81–70.85) a	1.07 ± 0.12	5.93	3	0.12
		Xinyang	720	2.63 (2.30–3.03) a	19.13 (13.50–31.28) a	1.91 ± 0.18	4.12	3	0.13
		Zhoukou	720	1.51 (1.28–1.77) bc	23.00 (15.02–41.62) a	1.39 ± 0.12	4.31	3	0.23
		Xinxiang	720	1.79 (1.50–2.16) b	21.51 (13.81–39.89) a	1.52 ± 0.14	4.15	3	0.13
		Ezhou	720	1.74 (1.47–2.08) b	30.21 (18.78–59.15) a	1.33 ± 0.12	1.29	3	0.73
DBN9501	DBN Vip3Aa	Xundian	576	0.14 (0.10–0.17) b	1.68 (1.10–3.23) a	1.51 ± 0.18	1.72	2	0.19
		Dehong	576	0.30 (0.25–0.37) a	3.73 (2.37–7.31) a	1.51 ± 0.16	1.17	2	0.28
		Puer	576	0.20 (0.11–0.30) ab	1.06 (0.57–7.52) ab	2.25 ± 0.29	4.79	2	0.09
		Xinyang	576	0.19 (0.17–0.22) b	0.57 (0.45–0.80) b	3.50 ± 0.37	1.95	2	0.16
		Zhoukou	576	0.30 (0.26–0.34) a	1.83 (1.38–2.67) a	2.08 ± 0.17	1.90	2	0.39
		Xinxiang	576	0.22 (0.13–0.35) ab	1.30 (0.67–10.37) ab	2.14 ± 0.27	4.67	2	0.10
		Ezhou	576	0.14 (0.12–0.15) b	0.44 (0.37–0.54) b	3.23 ± 0.29	2.93	2	0.23
DBN9936 × DBN9501	DBN Cry1Ab+Vip3Aa	Xundian	720	0.78 (0.61–0.96) c	14.15 (9.32–25.61) a	1.31 ± 0.13	4.27	3	0.12
		Dehong	720	1.38 (1.15–1.63) ab	24.45 (15.60–46.12) a	1.32 ± 0.12	1.79	3	0.62
		Puer	720	0.81 (0.63–1.01) c	17.84 (11.25–34.76) a	1.23 ± 0.12	3.60	3	0.17
		Xinyang	720	1.86 (1.55–2.23) a	26.95 (17.42–50.06) a	1.42 ±0.13	4.16	3	0.13
		Zhoukou	720	0.93 (0.77–1.09) bc	12.53 (8.82–20.29) a	1.46 ± 0.13	1.73	3	0.63
		Xinxiang	720	1.29 (1.08–1.52) b	20.40 (13.46–36.46) a	1.37 ± 0.12	3.52	3	0.32
		Ezhou	720	0.98 (0.78–1.20) bc	20.07 (12.67–38.73) a	1.25 ± 0.12	1.50	3	0.47
Bt11 × MIR162	Syngenta Cry1Ab+Vip3Aa	Xundian	720	0.36 (0.25–0.46) c	2.27 (1.85–3.04) b	2.05 ± 0.24	2.37	3	0.50
		Dehong	720	1.25 (0.83–1.71) ab	10.26 (6.01–29.05) a	1.80 ± 0.20	6.28	3	0.10
		Puer	720	0.63 (0.54–0.72) b	2.54 (2.14–3.19) b	2.72 ± 0.24	1.26	3	0.74
		Xinyang	720	1.42 (1.28–1.57) a	5.91 (4.97–7.33) a	2.66 ± 0.17	4.52	3	0.21
		Zhoukou	720	0.82 (0.72–0.92) b	3.20 (2.59–4.30) b	2.77 ± 0.25	0.49	3	0.78
		Xinxiang	720	1.01 (0.49–1.64) ab	5.56 (2.81–74.88) ab	2.23 ± 0.32	5.23	3	0.07
		Ezhou	720	0.43 (0.33–0.52) c	2.13 (1.76–2.77) b	2.37 ± 0.26	0.22	3	0.97

*N* = number of larvae in probit analysis. 95% FL = 95% fiducial limits. LC_50_ (LC_95_): concentration of protein (μg/g) required to kill 50% (95%) of larvae over 14 d. Values for the same protein and in the same column followed by the same lowercase letter did not differ significantly (overlapping 95% fiducial limits) in a χ^2^ test. *df* = degrees of freedom.

**Table 2:** Growth inhibitory concentrations of insecticidal proteins expressed in Bt maize against FAW larvae from different populations in China.

Bt Event	Protein	Population	*N*	GIC_50_ (95% FL) µg/g	GIC_95_ (95% FL) µg/g	Slope ± SE	χ^2^	*df*	*p*
DBN9936	DBN Cry1Ab	Xundian	720	0.38 (0.25–0.50) c	5.05 (3.56–8.59) ab	1.46 ± 0.17	0.48	3	0.92
		Dehong	720	1.04 (0.85–1.23) ab	6.58 (5.03–9.46) ab	2.05 ± 0.18	3.85	3	0.15
		Puer	720	0.63 (0.32–0.94) bc	4.04 (2.34–14.81) b	2.04 ± 0.30	7.77	3	0.06
		Xinyang	720	1.05 (0.94–1.18) ab	4.08 (3.37–5.22) b	2.80 ± 0.21	5.27	3	0.15
		Zhoukou	720	0.64 (0.52–0.76) b	4.62 (3.53–6.68) ab	1.92 ± 0.18	4.86	3	0.18
		Xinxiang	720	0.88 (0.73–1.03) b	7.38 (5.40–11.37) a	1.78 ± 0.16	5.02	3	0.17
		Ezhou	720	1.22 (1.08–1.39) a	5.82 (4.65–7.80) ab	2.43 ± 0.19	1.18	3	0.76
DBN9501	DBN Vip3Aa	Xundian	576	0.08 (0.06–0.10) d	0.35 (0.28–0.49) bc	2.66 ± 0.37	0.75	2	0.69
		Dehong	576	0.28 (0.25–0.32) a	0.88 (0.71–1.19) a	3.34 ± 0.38	0.06	2	0.81
		Puer	576	0.14 (0.12–0.16) bc	0.61 (0.48–0.85) ab	2.59 ± 0.28	1.23	2	0.54
		Xinyang	576	0.09 (0.06–0.11) cd	0.40 (0.32–0.56) b	2.48 ± 0.34	4.57	2	0.10
		Zhoukou	576	0.16 (0.14–0.18) b	0.53 (0.44–0.69) b	3.10 ± 0.31	4.56	2	0.10
		Xinxiang	576	0.14 (0.12–0.16) bc	0.47 (0.38–0.62) b	3.12 ± 0.33	0.26	2	0.88
		Ezhou	576	0.12 (0.11–0.13) c	0.25 (0.22–0.31) c	4.98 ± 0.58	1.46	2	0.48
DBN9936 × DBN9501	DBN Cry1Ab+Vip3Aa	Xundian	720	0.28 (0.03–0.50) b	2.14 (1.25–13.17) b	1.86 ± 0.37	6.90	3	0.08
		Dehong	720	0.43 (0.33–0.53) b	2.74 (2.15–3.88) b	2.05 ± 0.22	1.90	3	0.60
		Puer	720	0.54 (0.41–0.66) b	3.31 (2.57–4.65) ab	2.08 ± 0.21	3.60	3	0.17
		Xinyang	720	0.80 (0.68–0.94) a	3.34 (2.72–4.37) ab	2.66 ± 0.22	0.92	3	0.63
		Zhoukou	720	0.40 (0.30–0.51) b	3.10 (2.37–4.54) ab	1.86 ± 0.21	2.05	3	0.56
		Xinxiang	720	0.61 (0.47–0.74) ab	5.91 (4.30–9.37) a	1.66 ± 0.17	3.50	3	0.32
		Ezhou	720	0.87 (0.74–1.01) a	5.47 (4.23–7.76) a	2.06 ± 0.18	2.21	3	0.53
Bt11 × MIR162	Syngenta Cry1Ab+Vip3Aa	Xundian	720	0.24 (0.10–0.35) c	1.11 (0.90–1.60) b	2.46 ± 0.53	0.52	3	0.91
		Dehong	720	0.48 (0.35–0.59) bc	2.57 (2.05–3.61) a	2.25 ± 0.28	0.53	3	0.91
		Puer	720	0.55 (0.46–0.63) b	1.58 (1.33–2.03) b	3.59 ± 0.45	4.00	3	0.26
		Xinyang	720	0.78 (0.67–0.87) a	2.49 (2.09–3.18) a	3.25 ± 0.32	5.61	3	0.13
		Zhoukou	720	0.54 (0.42–0.64) b	2.14 (1.75–2.88) ab	2.74 ± 0.33	1.27	3	0.74
		Xinxiang	720	0.67 (0.56–0.77) ab	2.52 (2.07–3.33) a	2.85 ± 0.30	1.13	3	0.77
		Ezhou	720	0.39 (0.27–0.48) bc	1.32 (1.09–1.80) b	3.10 ± 0.51	0.04	3	0.98

*N* = number of larvae in the probit analysis. 95% FL = 95% fiducial limits. GIC_50_ (GIC_95_): growth inhibitory concentration of the protein (μg/g) required to cause 50% (95%) growth inhibition in the observation period of 14 d. Values for the same protein and in the same column followed by the same lowercase letter did not differ significantly (overlapping 95% fiducial limits) in a χ^2^ test. *df* = degrees of freedom.

**Table 3:** Mean (±SE) amount of two insecticidal Bt proteins produced in leaves of non-Bt maize lines and their associated Bt maize lines (in bold below the non-Bt line).

Maize Lines	Cry1Ab (µg·g^−1^)	Vip3Aa (µg·g^−1^)	Total Bt Protein (µg·g^−1^)
Nonghua 106	0.00	0.00	0.00
**DBN9936**	76.54 ± 0.60 b	0.00	76.54 ± 0.60 c
**DBN9501**	0.00	5.08 ± 0.08 c	5.08 ± 0.08 d
**DBN9936 × DBN9501**	74.51 ± 1.11 b	6.78 ± 0.13 b	81.29 ± 1.20 b
Xianda 901	0.00	0.00	0.00
**Bt11 × MIR162**	86.64 ± 1.01 a	24.83 ± 0.43 a	111.47 ± 1.17 a

Notes: See methods for extraction and quantification protocols. Values in the same column followed by different lowercase letters differed significantly (*p* < 0.05, Duncan’s test).

**Table 4:** Collection information for the studied FAW larval populations from maize fields in primary maize production areas in China.

Province	Location	Coordinates	Collection Date (2021)
Yunnan	Xundian	25.56° N, 103.26° E	28 August
Yunnan	Dehong	23.58° N, 97.48° E	25 November
Yunnan	Puer	22.68° N, 101.65° E	20 September
Henan	Xinyang	32.30° N, 114.07° E	3 July
Henan	Zhoukou	33.65° N, 114.69° E	20 October
Henan	Xinxiang	35.14° N, 113.77° E	14 October
Hubei	Ezhou	30.40° N, 114.89° E	17 September

## Data Availability

Not applicable.
